# Heart Rate Variability Dynamics in Padel Players: Set-by-Set and Rest Period Changes in Relation to Match Outcome

**DOI:** 10.3390/jfmk11010012

**Published:** 2025-12-26

**Authors:** Jon Mikel Picabea, Bingen Marcos-Rivero, Josu Ascondo, Javier Yanci, Cristina Granados

**Affiliations:** 1 Society, Sports and Physical Exercise Research Group (GIKAFIT), Physical Education and Sport Department, Faculty of Education and Sport, University of the Basque Country (UPV/EHU), 01007 Vitoria-Gasteiz, Spain; bingen.marcos@ehu.eus (B.M.-R.); josu.ascondo@ehu.eus (J.A.); javier.yanci@ehu.eus (J.Y.); 2 Research Group in Physical Activity, Exercise, and Sport (AKTIBOki), Department of Physical and Sports Education, Faculty of Education and Sport, University of the Basque Country (UPV/EHU), 01007 Vitoria-Gasteiz, Spain

**Keywords:** racket sport, fatigue, autonomic nervous system, competition, performance

## Abstract

**Objective**: The aim of this study was to analyse the evolution of heart rate variability (HRV) during official competition in high-level amateur padel players according to match outcome. **Methods**: HRV was measured in 44 individual recordings obtained across 11 matches involving 12 padel players. Measurements were taken before the match (PRE), during three sets (S1, S2 and S3) and during the two rest periods between sets (R1 and R2). Time-domain variables analysed included mean R–R interval (Mean RR), standard deviation of normalised R–R intervals (SDNN), root mean square of successive differences (RMSSD), natural logarithm of RMSSD (LnRMSSD) and standard deviation of heart rate (STD HR), while nonlinear variables included the transverse (SD1) and longitudinal (SD2) axes of the Poincare plot, stress score (SS) and the sympathetic–parasympathetic ratio (SNS/PNS ratio). **Results**: Significant fluctuations in HRV were observed throughout the match. Players who won exhibited significantly higher values of Mean RR, SDNN, RMSSD, LnRMSSD, SD1 and SD2 during S1 (*p* < 0.05), and higher Mean RR, RMSSD, LnRMSSD and SD1 during R1 (*p* < 0.01). These differences diminished as the match progressed, disappearing in the later phases (S3, R2). Temporal analysis revealed that both groups showed parasympathetic recovery during the rest periods. **Conclusions**: This study provides novel evidence on the temporal dynamics of autonomic regulation in padel, showing that match outcome is associated with differences in cardiovascular regulation during the initial phases of competition. These findings support the usefulness of HRV monitoring for performance management in real competition settings.

## 1. Introduction

Padel is a racket sport played in pairs (two vs. two) on a 20 × 10 metre court enclosed by walls or glass panels that allow the ball to rebound [1]. In recent years, this sport has undergone substantial growth in both the number of players and the availability of courts, and it is currently practised in more than 40 countries worldwide [1,2]. This increase in participation may be attributable to several advantages over similar racket sports that may have contributed to its rising popularity, such as its recreational and social nature, as it is played by four participants, as well as its accessibility, which enables individuals of all ages and ability levels to begin and enjoy the sport [2]. Moreover, padel is characterised by a high frequency of actions during play, which increases the pace and the number of rallies per point, while imposing lower physical and physiological demands than other racket sports [1,2,3,4].

The increase in the popularity of padel has been accompanied by a notable rise in scientific interest in this sport, with research mainly focusing on the analysis of performance indicators and the description of players’ activity during matches [1,2,5,6,7]. These studies have shown that padel is an intermittent sport that involves the participation of both the aerobic and anaerobic systems [8]. Specifically, it has been observed that the sport is predominantly aerobic, with a mean heart rate (Mean HR) of approximately 148 beats per minute [9]. However, the anaerobic system also plays an important role, particularly in explosive actions and rapid changes in direction executed during rallies [6]. Furthermore, psychological factors such as competitive anxiety, the continuous tactical decisions required in each action, and the need to perform technical skills with maximal precision also affect performance, due to the high cognitive demands and the cognitive and somatic anxiety and mental stress generated in padel [7,10]. Consequently, the combination of intermittent high-intensity efforts and the influence of psychological factors makes padel a sport with high physical and psychological demands, which affects the autonomic nervous system (ANS) [11,12,13]. For this reason, as has been done previously in racket sports [8,9,14] and in other individual and team sports [15,16,17], the analysis of heart rate variability (HRV) in competitive contexts could provide relevant information for a better understanding of this sport.

During exercise, increases in intensity predominantly activate the sympathetic nervous system (SNS) and inhibit the parasympathetic nervous system (PNS), leading to an increase in heart rate (HR) [18,19]. Heart rate variability (HRV) is a non-invasive tool that analyses fluctuations in the time between consecutive heartbeats through R–R intervals, thereby allowing the assessment of autonomic nervous system (ANS) activity and the determination of SNS and PNS activation levels [20,21]. HRV is measured using variables from three main domains: the time domain, the frequency domain and nonlinear variables [13,15]. However, frequency-domain variables appear to be sensitive to breathing patterns and to artefacts or noise, and it has therefore been recommended that HRV analysis be performed through time-domain and nonlinear variables [11,13,22]. Within the time domain, the most commonly used parameters are the root mean square of successive differences between R–R intervals (RMSSD) and the standard deviation of normalised consecutive R–R periods (SDNN) [23,24]. Both variables are influenced by fluctuations in HR, so the use of the natural logarithm of RMSSD (LnRMSSD) is recommended, as it reflects the beat-to-beat variance in HR and estimates changes in vagal activity [13]. Nonlinear HRV analysis enables evaluation of parasympathetic modulation without respiratory confounding. The principal parameters used are the transverse axis of the Poincaré plot (SD1), which represents parasympathetic cardiac activity, and the longitudinal axis of the Poincaré plot (SD2), which reflects the influence of both sympathetic and parasympathetic systems [13,25].

Although the study of heart rate variability (HRV) in padel has already been addressed in recent research, with significant decreases in HRV values observed after match play in high-level, intermediate-level and amateur players [8,9,26], it is still unknown how HRV evolves throughout the different sets of a match or during the various rest periods between them. Moreover, although some studies have examined the relationship between match outcome (winning or losing) and HRV in padel by assessing pre-match, in-match and post-match values [9,26], to the best of the authors’ knowledge, no investigations in racket sports have examined this issue by considering the natural or structural moments of play, such as sets and rest periods. Despite recent advances in HRV research in padel, existing studies have largely overlooked the analysis of intra-match autonomic dynamics across these structural phases, limiting our understanding of how autonomic regulation adapts during key moments of competition. Therefore, there is a clear need for studies that examine HRV responses within official match contexts and their association with match outcome, providing practical insights for performance and recovery management in racket sports.

Therefore, the aims of the present study were: (i) to analyse the evolution of heart rate variability (HRV) in padel players during official matches, both across different sets and in the various rest periods, and (ii) to determine whether differences in the evolution of HRV exist according to match outcome (winning or losing).

## 2. Materials and Methods

### 2.1. Participants

The present study analysed 44 individual HR recordings collected during the monitoring of 11 complete matches in a high-level amateur padel tournament. These recordings corresponded to 12 participating players who competed in a tournament that began with a group-stage phase, followed by a final competitive phase in which the top four pairs advanced to the semi-finals and the final. The variation in the number of recordings per player was due to their progression through the tournament rounds: four players were monitored twice, two players were monitored three times, five players were monitored four times and two players were monitored five times. Each recording reflects a player’s full participation in a match, allowing for a detailed analysis of physiological responses under real competitive conditions. The individual recordings were categorised into two groups based on match outcome: recordings from players who won (n = 22) and those who lost (n = 22). Inclusion criteria for a recording to be considered valid were that it covered the entire match and presented no interruptions or measurement errors. Only complete recordings from matches played during the tournament were included. Prior to the start of the study, all participants and the tournament organisers were informed about the objectives and procedures of the research. The study adhered to the guidelines of the Declaration of Helsinki [27] and was approved by the Ethics Committee for Research Involving Human Participants at the University of the Basque Country (UPV/EHU) (CEISH M10/2024/167).

### 2.2. Procedure

**Figure 1:**
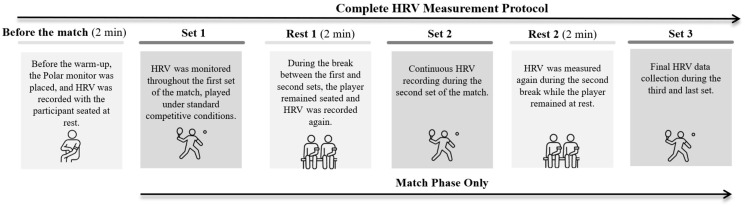
Measurement protocol for heart rate variability (HRV) during the different phases of a padel match.

HRV was measured at different points in the match, including pre-match (PRE), during each set (Set 1, Set 2, and Set 3), and during each rest period between sets (Rest 1 and Rest 2), in a high-level amateur padel tournament held indoors in February. The complete HRV measurement protocol, detailing the different recording moments during the matches, is presented in Figure 1. Prior to the start of each match, players performed their usual warm-up independently as part of their routine competition preparation.

### 2.3. Measurements

HRV analysis: Heart rate signals were recorded both during play and while players were seated before the match and during rest periods. Measurements were taken using a Polar OH1+ sensor (Kempele, Finland), which transmitted the data to a WIMU PROTM inertial device (Realtrack Systems, Almería, Spain) via Ant+ technology [28,29]. Both the seated and in-play measurements followed a protocol similar to that used in previous padel studies [26]. Seated measurements, including the pre-match recording and the recordings during rest periods, lasted 2 min, in accordance with the timing established by the tournament regulations.

The time-domain parameters obtained were as follows: (i) the mean R–R interval (Mean RR), (ii) the standard deviation of R–R intervals (SDNN), which reflects both sympathetic and parasympathetic alterations, (iii) the root mean square of successive differences between all R–R intervals (RMSSD), (iv) the natural logarithm of the root mean square of successive differences between all R–R intervals (LnRMSSD), and (v) the standard deviation of heart rate (HR STD) [13].

Regarding the nonlinear parameters analysed, the following were included: (i) the transverse axis of the Poincaré plot (SD1), which assesses short-term HRV and is an indicator of parasympathetic activity, (ii) the longitudinal axis of the Poincaré plot (SD2), which assesses long-term HRV and reflects both sympathetic and parasympathetic activity, (iii) the stress score, which indicates sympathetic changes and is calculated as 1000 × 1/SD2, and (iv) the SNS/PNS ratio, obtained using the formula SS/SD1 [13,30].

### 2.4. Statistical Analysis

Results are presented as mean ± standard deviation. Data were evaluated to verify normality and homogeneity of variance using the Shapiro–Wilk test and Levene’s test, respectively. To compare players who won and lost at the different moments of the match, independent samples Student’s *t* tests were applied for variables with normal distribution and equal variances, whereas the Mann–Whitney U test was used for variables that did not meet one or both assumptions. In addition, to simultaneously assess the effects of group (winning or losing) and match moment (S1, S2, S3 or PRE, R1, R2), a two-factor repeated measures ANOVA (2 × 3) was performed, followed by Bonferroni post hoc comparisons. As baseline differences between groups were identified in Set 1 for some variables, ANCOVA analyses were conducted in those cases, with initial values included as covariates, and Bonferroni post hoc tests applied. Effect sizes for pairwise differences were calculated using Cohen’s d for parametric variables and the rank-biserial correlation coefficient (*r_b_*) for non-parametric variables. The qualitative interpretation of effect sizes was as follows: d < 0.1 (no effect), 0.2–0.5 (small effect), 0.5–0.7 (intermediate effect) and 0.8–≥1.0 (large effect) [31]; and *r_b_* < 0.10 (very small), 0.10–0.29 (small), 0.30–0.49 (moderate) and ≥0.50 (large) [32]. All statistical analyses were conducted using JASP software (JASP for Windows, version 0.19, Amsterdam, The Netherlands). Statistical significance was set at *p* < 0.05.

## 3. Results

Table 1 presents the descriptive results and the evolution of HRV across the different moments of match play (S) for the total sample. Significant increases were observed in Mean RR between S1 and S2 (*p* < 0.01, *r_b_* = −0.51, large) and between S1 and S3 (*p* < 0.01, *r_b_* = −0.77, large). For SDNN, there was a significant decrease between S1 and S2 (*p* < 0.01, d = 3.06, large), followed by a significant increase between S2 and S3 (*p* < 0.01, d = 2.27, large). For RMSSD and LnRMSSD, significant increases were observed between S1 and S2 (*p* < 0.05, *r_b_* = 0.36 to 0.37, moderate). For STD HR, significant decreases were found between S1 and S2 (*p* < 0.01, d = −2.90, large) and between S1 and S3 (*p* < 0.01, d = −1.85, large). No significant differences between sets were observed for the remaining variables (SD1, SD2, SS, SNS/PNS ratio).

Table 2 presents the descriptive results and the evolution of HRV at the pre-match moment (PRE) and during the rest periods (R) between sets. Significant decreases were observed in Mean RR between PRE and R1 (*p* < 0.01, *r_b_* = 1.00, large) and between PRE and R2 (*p* < 0.01, *r_b_* = 0.79, large). For SDNN, significant decreases were found between PRE and R1 (*p* < 0.01, d = 1.15, large) and between PRE and R2 (*p* < 0.05, d = 0.61, intermediate). For RMSSD and LnRMSSD, significant decreases were observed between PRE and R1 (*p* < 0.01, d = −2.67 to −1.90, large), while a significant increase was observed between R1 and R2 (*p* < 0.01, *r_b_* = −1.00 to 1.87, large). For STD HR, significant decreases were observed from PRE to R1 (*p* < 0.01, d = 0.44, small). For SD1, significant decreases were observed between PRE and R1 (*p* < 0.01, d = −2.12, large) and between PRE and R2 (*p* < 0.01, d = 1.99, large), whereas significant increases were found between R1 and R2 (*p* < 0.01, d = 2.21, large). For SD2, significant decreases were observed between PRE and R1 (*p* < 0.01, d = 2.74, large), while significant increases were observed between R1 and R2 (*p* < 0.01, d = −1.55, large). Finally, for both SS and the SNS/PNS ratio, significant increases were observed between PRE and R1 (*p* < 0.01, d = −2.13 to 2.86, large) and between R1 and R2 (*p* < 0.01, *r_b_* = −1.00 to 0.77, large).

Table 3 presents the HRV results across the different sets of the match according to match outcome (winning or losing). When the sets were compared based on match result, significantly higher values were observed in the winning group in S1 for Mean RR (*p* < 0.05, *r_b_* = −0.50, intermediate), SDNN (*p* < 0.01, d = −0.37, small), RMSSD (*p* < 0.01, *r_b_* = −0.66, large), LnRMSSD (*p* < 0.05, *r_b_* = −0.68, large), SD1 (*p* < 0.05, *r_b_* = −0.37, moderate) and SD2 (*p* < 0.05, d = −0.37, moderate). In contrast, no statistically significant differences were observed between winners and losers in S2 or S3 for any of the HRV variables analysed.

Regarding the temporal evolution within each group, significant changes were identified across sets. In the winning group, increases were observed in Mean RR between S1 and S2 (*p* < 0.05, d = −0.44, small) and between S1 and S3 (*p* < 0.05, d = −0.77, large). Decreases in SDNN were observed between S1 and S2 (*p* < 0.01, *r_b_* = 1.00, large), followed by an increase between S2 and S3 (*p* < 0.01, d = −2.03, large). Reductions in STD HR were found between S1 and S2 (*p* < 0.01, d = −2.89, large) and between S1 and S3 (*p* < 0.01, d = −0.96, large). Increases between S1 and S2 were observed for RMSSD (*p* < 0.01, d = −0.67, intermediate), LnRMSSD (*p* < 0.01, d = −0.76, large) and SD1 (*p* < 0.01, d = 0.52, intermediate). In the losing group, significant increases in Mean RR were observed between S1 and S2 (*p* < 0.05, *r_b_* = −0.53, large) and between S1 and S3 (*p* < 0.01, *r_b_* = −0.89, large). Increases in SDNN were found between S1 and S2 (*p* < 0.01, d = 2.75, large) and between S2 and S3 (*p* < 0.01, d = −2.62, large). Increases in STD HR were observed between S1 and S2 (*p* < 0.01, d = −2.91, large) and between S1 and S3 (*p* < 0.01, d = −2.27, large).

For the variables that did not show baseline differences in S1 (STD HR, SS, SNS/PNS ratio), the two-factor repeated measures ANOVA revealed significant differences in the interaction between group and time for STD HR (*p* < 0.001). In contrast, for the variables that did show baseline differences in S1, the ANCOVA analyses indicated no significant effects of match outcome on the group-by-time interaction (*p* > 0.05).

Similarly, Table 4 presents the HRV results obtained during the different rest moments of the match according to match outcome (winning or losing). When comparing groups at each time point, significantly higher values were observed in the winning group during R1 for Mean RR (*p* < 0.01, *r_b_* = −0.86, large), RMSSD (*p* < 0.01, *r_b_* = −0.89, large), LnRMSSD (*p* < 0.01, *r_b_* = −0.97, large) and SD1 (*p* < 0.01, *r_b_* = −0.89, large). No significant differences between groups were found in PRE or R2 for any of the variables analysed.

Regarding the temporal evolution within each group, significant changes were identified from PRE to R1 in both groups. Specifically, in the winning group, significant decreases were observed in Mean RR (*p* < 0.01, d = 1.00, large), SDNN (*p* < 0.01, d = 1.02, large), RMSSD (*p* < 0.01, d = 1.93, large), LnRMSSD (*p* < 0.01, *r_b_* = −2.08, large), SD1 (*p* < 0.01, *r_b_* = −1.97, large), SD2 (*p* < 0.01, d = 3.09, large) and STD HR (*p* < 0.01, d = 0.69, intermediate), while significant increases were observed in SS (*p* < 0.01, d = 3.83, large) and the SNS/PNS ratio (*p* < 0.01, *r_b_* = −3.08, large). In the losing group, significant decreases were found in Mean RR (*p* < 0.01, d = 1.67, large), SDNN (*p* < 0.01, d = 1.32, large), RMSSD (*p* < 0.01, d = 1.54, large), LnRMSSD (*p* < 0.01, *r_b_* = −1.70, large), SD1 (*p* < 0.01, *r_b_* = −2.35, large) and SD2 (*p* < 0.01, d = 2.68, large), together with significant increases in SS (*p* < 0.01, d = 2.51, large) and the SNS/PNS ratio (*p* < 0.01, *r_b_* = −1.56, large). Between PRE and R2, winners showed significant decreases in Mean RR (*p* < 0.01, d = 1.38, large), SDNN (*p* < 0.05, d = 0.77, large) and SD1 (*p* < 0.01, d = 2.81, large), whereas losers showed significant decreases only in SD1 (*p* < 0.01, d = 1.50, large). In the comparison between R1 and R2, the winning group showed a significant decrease in Mean RR (*p* < 0.05, d = 0.79, intermediate) and significant increases in RMSSD (*p* < 0.01, d = −1.00, large), LnRMSSD (*p* < 0.01, *r_b_* = 1.00, large), SD1 (*p* < 0.01, *r_b_* = 1.00, large), SD2 (*p* < 0.01, d = −1.53, large) and SS (*p* < 0.01, *r_b_* = −1.00, large). The losing group showed significant increases in RMSSD (*p* < 0.01, d = −1.40, large), LnRMSSD (*p* < 0.01, *r_b_* = 1.49, large), SD1 (*p* < 0.01, *r_b_* = 1.85, large), SD2 (*p* < 0.01, d = −1.48, large), SS (*p* < 0.01, *r_b_* = −1.66, large) and the SNS/PNS ratio (*p* < 0.05, *r_b_* = 1.18, large).

The two-factor repeated measures ANOVA revealed significant temporal effects for Mean RR (*p* < 0.001), SDNN (*p* = 0.003), RMSSD (*p* < 0.001), LnRMSSD (*p* < 0.001), SD1 (*p* < 0.001), SD2 (*p* < 0.001) and the SNS/PNS ratio (*p* < 0.001). No significant group-by-time interactions were found for any of the variables analysed.

## 4. Discussion

The present study aimed to analyse HRV in padel players both in relation to match outcome and to its evolution throughout competition. The results indicate a progressive activation of the ANS during active moments of play and a partial recovery during rest periods, with physiological responses differing according to match outcome. Furthermore, a significant temporal effect was observed in most variables, as well as a significant interaction between match outcome and match moment for STD HR during playing periods. These differences may be mediated by psychological factors such as motivation and confidence, which vary according to the course of the match and directly affect HRV [7,10].

### 4.1. Evolution of Physiological Responses Throughout the Match for All Players

The results of this study showed a dynamic evolution of HRV throughout the match, reflecting distinct physiological adaptations between playing periods and rest intervals. During the sets, a significant and progressive increase in Mean RR was observed from S1 to S3. SDNN decreased significantly from S1 to S2, followed by a significant increase from S2 to S3. Similarly, RMSSD and LnRMSSD displayed significant increases between S1 and S2, whereas STD HR decreased significantly from S1 to the subsequent sets. During the rest periods between sets, comparison with PRE revealed significant decreases in Mean RR, SDNN, RMSSD, LnRMSSD, SD1 and SD2 after the first set (R1). However, comparison between rest intervals indicated partial recovery, with significant increases from R1 to R2 in RMSSD, LnRMSSD, SD1 and SD2. In parallel, the sympathetic stress markers SS and the SNS/PNS ratio showed significant increases both from PRE to R1 and from R1 to R2.

These findings are consistent with previous literature reporting significant competition-induced alterations in HRV, indicative of accumulated physiological stress, both in padel [8,9,26] and in other racket sports [15,33,34]. However, the present study provides a deeper perspective by quantifying this evolution in a structured manner throughout the natural phases of the match. For example, in the same sport, Parraca et al. [8] observed a significant decrease in RMSSD during match play with partial increases during recovery periods, which aligns with our results. Nevertheless, the findings of the present study show that this recovery varies across phases of the match, being significant between S1 and S2 but not between S1 and S3. Other studies [15,26] have reported generalised decreases in multiple HRV variables (RMSSD, SD1, SD2, Mean RR, SDNN, LnRMSSD) after competition, corroborating the state of acute fatigue induced by exertion. Likewise, in elite badminton players [35], significant post-competition reductions in HRV were observed, which correlated with higher perceived fatigue, highlighting the utility of HRV as an indicator of ANS status and recovery levels. The distinction between these previous studies and the present work lies in the intra-match analysis of HRV within a competitive context that incorporates the natural phases of match play. This approach provides a more precise understanding of the actual demands of competition, offering valuable insights for planning recovery and preparation strategies in padel.

### 4.2. Differences in Physiological Responses According to Match Outcome

It should be noted that, although physiological responses during padel competition have been previously analysed, particularly in relation to cardiovascular load, external demands and fatigue responses [36], the study of HRV in this sport remains limited. The results showed that differences in HRV between winners and losers were not uniform throughout the match but were primarily concentrated in the initial phases. Specifically, players who won displayed significantly higher values in S1 for Mean RR, SDNN, RMSSD, LnRMSSD, SD1 and SD2, suggesting greater parasympathetic activation and more efficient autonomic control at the beginning of play. This pattern was also evident in R1, where winners again exhibited higher values in Mean RR, RMSSD, LnRMSSD and SD1. However, these differences were not present in PRE and did not persist in the later phases of the match. Only Mean RR differed between groups during S2, and these differences disappeared entirely in S3 and R2. These findings partially align with previous research. Villafaina et al. [9], for instance, analysed HRV during friendly padel matches and hypothesised that losing players would present lower HRV. However, they did not observe significant differences based on match outcome, suggesting that physical load and physiological stress were similar in both groups, which contrasts with the results of the present study. In line with these findings, Conde-Ripoll et al. [26] also did not observe differences in HRV between winners and losers. However, they reported that losing players had significantly higher mean, minimum, and maximum heart rates during the match. Similarly, a study in table tennis under simulated competition conditions observed no significant differences in time-domain or nonlinear HRV variables according to match outcome [12], although differences were found in frequency-domain variables. However, some studies have reported differences between players who won and those who lost. For example, greater match demands have been observed among losing padel players [37], consistent with the present study’s results. Specifically, Roldán-Márquez et al. [37] found in official padel competition that players who lost exhibited more intense physiological responses (higher heart rate, greater distance covered and a higher number of sprints) than players who won, suggesting greater ANS activation in situations of increased competitive demand. The discrepancy between previous studies and the findings of the present work may indicate that competitive context influences HRV responses according to match outcome, which could explain the differences observed in this study. In this regard, HRV may be considered an indirect marker of competitive efficiency, as lower ANS activation has been associated with greater autonomic control and lower perceived effort, potentially facilitating decision-making under pressure, resilience to competitive stress and more efficient recovery between points or sets [13,38].

### 4.3. Evolution of Physiological Responses During the Match According to Match Outcome

The analysis of HRV temporal evolution revealed distinct patterns between winners and losers across different moments of the match. Players who won showed a progressive improvement in Mean RR, with increases from S1 to S2 and from S1 to S3, together with early vagal recovery, reflected in increases in RMSSD, LnRMSSD and SD1 between S1 and S2. During the rest periods, this group demonstrated parasympathetic recovery, with significant increases in RMSSD, LnRMSSD, SD1 and SD2 from R1 to R2. In contrast, although players who lost also showed recovery during the rest periods in these same variables, they started from lower baseline levels in S1 and exhibited a more irregular pattern during play, with marked fluctuations in SDNN, characterised by decreases from S1 to S2 followed by an increase from S2 to S3. Additionally, both groups experienced sustained sympathetic activation during the rest periods, reflected in significant increases in SS and the SNS/PNS ratio from PRE to R1 and from R1 to R2. Moreover, once baseline differences were controlled for, the temporal evolution of HRV was similar between groups for most variables, with no significant interaction effects, except for STD HR during match play, where a significant interaction was observed between match outcome (winning/losing) and temporal moment (sets/rest periods).

In this regard, the results observed in the winning group are consistent with those reported in padel by Parraca et al. [9], in which RMSSD exhibited a U-shaped response, with a decrease during exercise followed by recovery, in line with previous studies [13,39]. However, Parraca et al. [9] did not find significant differences in HRV across the different moments analysed during the match (minutes 30, 60 and 90). This contrasts with the findings of the present study, in which significant temporal differences were observed. Such discrepancies may be attributed to methodological differences, as Parraca et al. [9] used predetermined time intervals, whereas the present study examined the structural phases of match play (sets and rest periods). Accordingly, the results of the present study showed a more irregular regulatory pattern in losing players, characterised by marked fluctuations in SDNN and smaller progressive increases in Mean RR, whereas in Parraca et al. [9] autonomic modulation appeared more stable. Nevertheless, this comparison has notable limitations, as the present study analysed match evolution using the structural phases of official competition (pre-match, sets and rest periods), which provide a more accurate representation of the actual dynamics of exertion and recovery. Overall, these findings suggest that HRV varies according to match outcome, although further studies are needed to examine its progression in official competition while considering the natural phases of match play.

Although this study was conducted with high scientific rigour, it is not without limitations. The main limitation is the scarcity of previous research that jointly analyses match outcome and the temporal moments of play, which limited the possibility of direct comparison with other studies. Additionally, the absence of post-match measurements prevented the assessment of full ANS recovery after the match, allowing only the evaluation of intra-match recovery. Moreover, several uncontrolled variables in this study may have influenced HRV and, consequently, the results obtained, such as physical load, psychological stress or the quality of sleep the night before the match. Therefore, it would be advisable for future research to include a more diverse sample, considering different competitive levels and the natural temporal phases of the match, as well as incorporating post-match measurements to enable a more comprehensive follow-up of recovery, together with the control of additional variables that may affect HRV.

## 5. Conclusions

The results of the present study demonstrate a dynamic and complex evolution of HRV during official padel competition, characterised by sympathetic activation and altered autonomic control, accompanied by partial parasympathetic recovery during rest periods. The main difference related to match outcome was that players who won displayed a more favourable autonomic profile in the initial phases of the match, with significantly higher values in the first set and the first rest period. Although both groups showed recovery mechanisms, the winners exhibited a more efficient and stable regulatory pattern throughout the match. These findings provide relevant information on the actual physiological demands of padel during official competition, which may help optimise the planning of training and recovery strategies, thereby supporting improved performance in this sport.

## Figures and Tables

**Table 1:** Results of heart rate variability (HRV) variables across the different sets of the match for the entire sample analysed.

Variables	S1	S2	S3	ES S1–S2	ES S1–S3	ES S2–S3
Mean RR (ms)	385.98 ± 70.27	403.37 ± 36.86	422.22 ± 34.27	−0.51 **	−0.77 **	*−0.37*
SDNN (ms)	38.38 ± 11.42	37.58 ± 8.98	41.93 ± 15.27	*3.06* **	−0.13	*2.27* **
RMSSD (ms)	2.43 ± 0.57	2.62 ± 0.52	2.93 ± 0.45	0.37 *	0.25	*0.11*
LnRMSSD	0.87 ± 0.22	0.94 ± 0.20	1.06 ± 0.16	0.36 *	*0.05*	0.11
STD HR (ms)	13.83 ± 3.49	13.29 ± 2.81	13.59 ± 4.83	*−2.90* **	*−1.85* **	−0.20
SD1 (ms)	1.72 ± 0.40	1.86 ± 0.37	2.07 ± 0.32	0.19	0.10	*0.13*
SD2 (ms)	54.25 ± 16.15	53.11 ± 12.70	59.24 ± 21.67	−0.12	−0.39	−0.26
SS	18.64 ± 5.47	19.86 ± 4.70	31.23 ± 59.80	*0.25*	0.15	0.22
SNS/PNS ratio	11.21 ± 4.98	11.49 ± 5.09	15.47 ± 28.87	0.13	0.08	0.05

Notes: Mean RR: Mean R–R interval; SDNN: Standard deviation of normalised R–R intervals; RMSSD: Root mean square of successive differences; LnRMSSD: Natural logarithm of RMSSD; STD HR: Standard deviation of heart rate; SD1: Transverse axis of the Poincaré plot; SD2: Longitudinal axis of the Poincaré plot; SS: Stress score; SNS/PNS ratio: Sympathetic–parasympathetic ratio; S = Set; ES = Effect size; ** *p* < 0.01; * *p* < 0.05. *Italics* = Parametric.

**Table 2:** Results of heart rate variability (HRV) variables in the pre-match moment and during rest periods (R) for the entire sample analysed.

Variables	PRE	R1	R2	ES PRE–R1	ES PRE–R2	ES R1–R2
Mean RR (ms)	566.31 ± 148.49	409.63 ± 77.64	432.54 ± 41.68	1.00 **	0.79 **	0.14
SDNN (ms)	67.04 ± 28.62	35.60 ± 12.69	39.09 ± 18.79	*1.15* **	*0.61* *	*−0.03*
RMSSD (ms)	5.01 ± 2.31	2.76 ± 0.88	3.27 ± 1.19	*−2.67* **	−0.52	−1.00 **
LnRMSSD	1.52 ± 0.48	0.98 ± 0.31	1.13 ± 0.33	*−1.90* **	−0.42	*1.87* **
STD HR (ms)	12.82 ± 6.26	12.20 ± 4.35	12.71 ± 6.27	*0.44* **	0.31	−0.08
SD1 (ms)	3.50 ± 1.56	1.90 ± 0.63	2.26 ± 0.85	*−2.12* **	*1.99* **	*2.21* **
SD2 (ms)	94.73 ± 40.47	50.31 ± 17.95	55.20 ± 26.63	*2.74* **	0.09	*−1.55* **
SS	10.78 ± 4.46	21.16 ± 8.24	34.88 ± 59.63	*2.86* **	−0.46	−1.00 **
SNS/PNS ratio	3.43 ± 2.38	12.42 ± 7.97	18.02 ± 29.39	*−2.13* **	−0.39	0.77 **

Notes: Mean RR: Mean R–R interval; SDNN: Standard deviation of normalised R–R intervals; RMSSD: Root mean square of successive differences; LnRMSSD: Natural logarithm of RMSSD; STD HR: Standard deviation of heart rate; SD1: Transverse axis of the Poincaré plot; SD2: Longitudinal axis of the Poincaré plot; SS: Stress score; SNS/PNS ratio: Sympathetic–parasympathetic ratio; R = Rest; PRE = Pre-match; ES = Effect size; ** *p* < 0.01; * *p* < 0.05. *Italics* = Parametric.

**Table 3:** Results of heart rate variability (HRV) variables across the different sets of the match according to match outcome.

Variables	S1	S2	S3	ES S1–S2	ES S1–S3	ES S2–S3
Mean RR (ms)						
Win	408.04 ± 31.81	414.64 ± 30.62	426.72 ± 29.92	*−0.44* *	*−0.77* *	*−0.23*
Lose	358.11 ± 93.60	389.12 ± 39.86	416.60 ± 40.45	−0.53 *	−0.89 *	−0.54
ES	−0.50 **	*−0.73* **	*−0.29*			
SDNN (ms)						
Win	42.03 ± 10.27	37.80 ± 7.09	38.18 ± 15.48	1.00 **	*0.49*	*−2.03* **
Lose	33.78 ± 11.38	37.30 ± 11.12	46.63 ± 14.59	*2.75* **	*−0.62*	*−2.62* **
ES	*−0.37* **	*−0.14*	*0.20*			
RMSSD (ms)						
Win	2.59 ± 0.41	2.68 ± 0.44	2.94 ± 0.41	*0.67* **	*0.71*	*0.37*
Lose	2.23 ± 0.67	2.55 ± 0.61	2.91 ± 0.53	−0.17	−0.28	−0.10
ES	−0.66*	*−0.24*	*−0.07*			
LnRMSSD						
Win	0.94 ± 0.17	0.97 ± 0.17	1.07 ± 0.14	*−0.76* **	*0.72*	*0.41*
Lose	0.80 ± 0.26	0.91 ± 0.24	1.05 ± 0.19	−0.19	−0.28	*−0.10*
ES	−0.68 *	*−0.30*	*−0.12*			
STD HR (ms)						
Win	14.21 ± 2.93	12.85 ± 2.78	11.99 ± 4.70	*−2.89* **	−0.96 **	0.30
Lose	13.34 ± 4.12	13.85 ± 2.82	15.59 ± 4.47 ***	*−2.91* **	*−2.27* **	*−0.69*
ES	*−0.07*	*0.21*	*0.45*			
SD1 (ms)						
Win	1.84 ± 0.29	1.90 ± 0.31	2.09 ± 0.29	*0.52* *	*0.53*	*0.36*
Lose	1.58 ± 0.48	1.81 ± 0.43	2.06 ± 0.38	−0.28	−0.50	*−0.06*
ES	−0.37 *	*−0.13*	*0.00*			
SD2 (ms)						
Win	59.41 ± 14.52	53.42 ± 10.03	53.91 ± 22.01	*0.01*	−0.16	0.13
Lose	47.74 ± 16.08	52.72 ± 15.72	65.90 ± 20.64	*−0.33*	−0.61	*−0.69*
ES	*−0.37* *	*−0.14*	*0.20*			
SS						
Win	17.69 ± 3.79	19.42 ± 4.07	42.94 ± 79.93	*0.16*	0.13	−0.20
Lose	19.84 ± 6.99	20.41 ± 5.46	16.60 ± 5.31	*0.27*	*0.09*	*0.78*
ES	*0.40*	*0.21*	−0.44			
SNS/PNS ratio						
Win	10.13 ± 3.66	10.64 ± 3.29	20.98 ± 38.55	*−0.15*	−0.20	−0.38
Lose	12.57 ± 6.10	12.57 ± 6.66	8.57 ± 3.90	*0.38*	*0.37*	*0.76*
ES	*0.27*	*0.13*	−0.22			

Notes: Mean RR: Mean R–R interval; SDNN: Standard deviation of normalised R–R intervals; RMSSD: Root mean square of successive differences; LnRMSSD: Natural logarithm of RMSSD; STD HR: Standard deviation of heart rate; SD1: Transverse axis of the Poincaré plot; SD2: Longitudinal axis of the Poincaré plot; SS: Stress score; SNS/PNS ratio: Sympathetic–parasympathetic ratio; S = Set; ES = Effect size; *** *p* < 0.001; ** *p* < 0.01; * *p* < 0.05. *Italics* = Parametric.

**Table 4:** Results of heart rate variability (HRV) variables in the pre-match moment and during rest periods (R) according to match outcome.

Variables	PRE	R1	R2	ES PRE–R1	ES PRE–R2	ES R1–R2
Mean RR (ms)						
Win	583.32 ± 137.20	437.12 ± 36.91	434.36 ± 39.38	1.00 **	*1.38* **	*0.79* *
Lose	544.83 ± 162.86	374.91 ± 100.22	430.27 ± 47.07	*1.67* **	*0.56*	−0.67
ES	−0.07	−0.86 **	*−0.10*			
SDNN (ms)						
Win	68.79 ± 31.65	37.91 ± 10.10	39.70 ± 20.06	*1.02* **	*0.77* *	*0.07*
Lose	64.82 ± 24.94	32.69 ± 15.15	38.32 ± 18.41	*1.32* **	0.41	*−0.13*
ES	*−0.15*	*−0.39*	*−0.07*			
RMSSD (ms)						
Win	4.93 ± 2.47	3.08 ± 0.77	3.18 ± 1.24	*1.93* **	−0.27	−1.00 **
Lose	5.11 ± 2.16	2.36 ± 0.87	3.38 ± 1.21	*1.54* **	*−0.76*	*−1.40* **
ES	*0.08*	−0.89 **	*0.16*			
LnRMSSD						
Win	1.50 ± 0.49	1.10 ± 0.22	1.11 ± 0.30	*−2.08* **	−0.22	1.00 **
Lose	1.55 ± 0.48	0.83 ± 0.34	1.16 ± 0.40	*−1.70* **	*−0.67*	*1.49* **
ES	*0.12*	−0.97 **	*0.14*			
STD HR (ms)						
Win	12.62 ± 5.55	12.08 ± 3.34	12.80 ± 7.05	*0.69* **	0.49	−0.42
Lose	13.06 ± 7.21	12.35 ± 5.46	12.60 ± 5.61	*0.23*	*−0.09*	0.33
ES	*0.07*	*0.06*	*−0.03*			
SD1 (ms)						
Win	3.45 ± 1.67	2.14 ± 0.56	2.19 ± 0.89	*−1.97* **	*2.81* **	1.00 **
Lose	3.56 ± 1.45	1.61 ± 0.61	2.35 ± 0.86	*−2.35* **	*1.50* **	*1.85* **
ES	*0.11*	−0.89 **	*0.19*			
SD2 (ms)						
Win	97.22 ± 44.75	53.56 ± 14.28	56.05 ± 28.46	*3.09* **	0.35	*−1.53* **
Lose	91.59 ± 35.28	46.20 ± 21.43	54.13 ± 26.04	*2.68* **	*−0.24*	*−1.48* **
ES	*−0.15*	*−0.39*	*−0.07*			
SS						
Win	10.80 ± 4.49	20.17 ± 6.36	43.67 ± 79.89	*3.83* **	−0.53	−1.00 **
Lose	10.75 ± 4.54	22.41 ± 10.18	23.89 ± 13.47	*2.51* **	*−0.51*	*−1.66* **
ES	*−0.01*	*0.27*	−0.33			
SNS/PNS ratio						
Win	3.70 ± 2.82	10.12 ± 4.29	21.76 ± 38.49	*−3.08* **	−0.56	0.55
Lose	3.09 ± 1.67	15.31 ± 10.43	13.34 ± 12.13	*−1.56* **	*−0.31*	*1.18* *
ES	*−0.16*	*0.27*	−0.28			

Notes: Mean RR: Mean R–R interval; SDNN: Standard deviation of normalised R–R intervals; RMSSD: Root mean square of successive differences; LnRMSSD: Natural logarithm of RMSSD; STD HR: Standard deviation of heart rate; SD1: Transverse axis of the Poincaré plot; SD2: Longitudinal axis of the Poincaré plot; SS: Stress score; SNS/PNS ratio: Sympathetic–parasympathetic ratio; R = Rest; PRE = Pre-match; ES = Effect size; ** *p* < 0.01; * *p* < 0.05. *Italics* = Parametric.

## Data Availability

The original contributions presented in this study are included in the article. Further inquiries can be directed to the corresponding authors.

## Abbreviations

The following abbreviations are used in this manuscript:

HR	Heart rate
HRV	Heart rate variability
ANS	Autonomic nervous system
SNS	Sympathetic nervous system
PNS	Parasympathetic nervous system
PRE	Pre-match
S1, S2, S3	Set 1, Set 2, Set 3
R1, R2	Rest period 1, Rest period 2
Mean RR	Mean R–R interval
SDNN	Standard deviation of normalised R–R intervals
RMSSD	Root mean square of successive differences
LnRMSSD	Natural logarithm of RMSSD
STD HR	Standard deviation of heart rate
SD1	Transverse axis of the Poincaré plot
SD2	Longitudinal axis of the Poincaré plot
SS	Stress score
SNS/PNS ratio	Sympathetic–parasympathetic ratio
ES	Effect Size
